# Development and Application of an SNP Marker for High-Throughput Detection and Utilization of the *badh2* Gene in Rice Breeding

**DOI:** 10.3390/genes16101132

**Published:** 2025-09-25

**Authors:** Hao Fang, Huifang Huang, Lan Yu, Linyou Wang, Jue Lou, Yongbin Qi

**Affiliations:** 1 Institute of Rural Development, Zhejiang Academy of Agricultural Sciences, Hangzhou 310021, China; pujiangfanghao@163.com; 2 Hangzhou Agricultural Technology Extension Center, Hangzhou 310020, China; huang02fang@21cn.com; 3 Pujiang County Improved Seed Breeding Center, Jinhua 322299, China; 18905892080@163.com; 4 Institute of Crop Science and Nuclear Technology Utilization, Zhejiang Academy of Agricultural Sciences, Hangzhou 310021, China; ly217wang@sina.com; 5 Southern Zhejiang Key Laboratory of Crop Breeding, Wenzhou Vocational College of Science and Technology, Wenzhou 325006, China

**Keywords:** aromatic rice, *badh2-E2*, *badh2-E7*, multiplex-ready PCR, KASP

## Abstract

**Background:** As a key rice breeding resource, aromatic rice is widely cultivated in agriculture due to its unique aroma. *Badh2* mutations cause function loss, enabling rice’s characteristic aroma. **Methods**: In this study, we analyzed several *badh2* mutation types across 8 *japonica* and 16 *indica* aromatic rice lines. Based on the 7 bp deletion in *badh2-E2* identified in *japonica* aromatic lines, we developed a multiplex-ready PCR assay for *badh2* genotyping. Additionally, leveraging the deletion mutation in *badh2-E7* from the *indica* aromatic line Yexiang, we designed a KASP marker. **Results**: All 8 *japonica* aromatic lines carried a 7 bp deletion in *badh2-E2*, while 12 *indica* aromatic lines harbored an 8 bp deletion in *badh2-E7*, and 4 additional *indica* aromatic lines exhibited an 8 bp deletion in *badh2-E2*. The multiplex-ready PCR assay was used to screen 200 individual plants from the aromatic rice line Jia 58: 199 plants showed the expected results, while the remaining 1 exhibited two fluorescent signal peaks—suggesting that it may be a heterozygous individual. Using the KASP marker, we performed genotyping analysis on F_7_ progeny individuals derived from the cross between Yexiang (aromatic line) and Yuenongsimiao (non-aromatic line). Combined with phenotypic observations, we successfully screened out an elite aromatic line named Zhexiangzhenhe, which not only possesses aroma but also maintains superior agronomic traits. **Conclusions**: The multiplex-ready PCR assay and KASP markers facilitate high-throughput genotyping in large-scale breeding populations, providing breeders with a rapid and efficient selection tool to accelerate aromatic trait improvement in rice.

## 1. Introduction

Rice is the most important stamp crop in the world, especially in China and South-east Asia. Rice grains are a main source of income for farmers. Due to its special aroma and good market value, the demand for aromatic rice has been increasing in recent years. In order to improve the breeding efficiency of aromatic rice varieties, identifying an inexpensive, simple, and high-throughput marker for aromatic rice is becoming increasingly important for rice farmers. The farmers can use the marker to quickly select the ideal individual plant that not only has high yield and good resistance to stress but also aromatic smell.

Cultivating an aromatic rice variety is the most valuable aspect of rice breeding, as farmers can earn more from planting aromatic rice. According to previous studies, 2-acetyl-1-pyrroline (2-AP) levels play a crucial role in the grains of aromatic rice, despite detecting one hundred compounds in them [1,2,3,4]. The 2-AP concentration in aromatic rice grains is higher than non-aromatic ones and can be detected by human olfactory organs [5,6]. Presently, there are two methods of detecting flavor or aroma in rice grains: a subjective method and an objective method. Discriminating between the aromatic and non-aromatic varieties using the subjective method mainly depends on the analysts’ taste of individual grains or the smell of the leaf tissue or grain that has been heated in water or reacted with KOH or I_2_/KI solutions [7]. Objective detection uses gas chromatography to measure the quantity of rice grains in rice and distinguish the rice varieties [8,9]. However, the subjective method needs a large amount of man power. Subjective detection accuracy is still not very reliable. The objective method requires expensive instruments and equipment and has high test costs. Given the limitation of these methods and the development, identification, and cloning of rice aromatic genes, marker-assisted selection appears to have more advantages than subjective and objective detection in rice breeding. Marker-assisted selection allows for the rapid selection of individual plants with aromatic traits by segregating generations, and these traits can be quickly stabilized [10,11]. Moreover, it is not affected by environmental conditions, making it a simple and efficient selection method [12].

In a previous study, *badh2*, a single recessive gene, which is located on chromosome 8 of rice, was reported to contain 14 introns and 15 exons, and it is the main gene that encodes betaine aldehyde dehydrogenase (*Badh2*), which is associated with rice aroma. Fragment deletion on exons 2 and 7 will lead to the function loss of *badh2* and inhibits 2-acetyl-1-pyrroline (2-AP) synthesis, which produces aroma in rice grains [13,14,15,16,17]. A small fragment deletion can be used as an SNP marker in molecular marker-assisted breeding programs for rice. SNP markers have been successfully developed and used in aromatic rice breeding [18]. In addition, several PCR-based molecular markers have been developed to detect the *badh2* gene in rice. With the continuous discovery and identification of *badh2* gene haplotypes, molecular markers based on the characteristics of these haplotypes have been continuously developed and applied in marker-assisted breeding, enabling breeders to rapidly improve the aromatic traits of rice [14,19,20,21,22]. Presently, different kinds of methods for SNP detection were used in molecular breeding, including allele-specific PCR, cleaved amplified polymorphism sequences (CAPSs), derived CAPSs (dCAPSs), high-resolution melting (HRM), temperature switch PCR (TS-PCR), and Kompetitive allele-specific PCR (KASP) [23]. As KASP has many advantages, including high throughput, high speed and accuracy, good stability, and low cost, it has been widely used in marker-assistant selection, bulked segregation analysis, and single nucleotide polymorphism (SNP) genotyping [23]. As a new method for SNP genotyping, multiplex-ready PCR can be widely applied in marker-assisted breeding, and it is particularly effective in identifying and utilizing small-fragment insertion–deletion (Indel) polymorphisms. In particular, for cases where common PCR amplification methods cannot be used to design primers for genotyping analysis due to the high GC content of DNA fragments near SNP loci, primers can be designed using DNA regions with low GC content upstream and downstream of SNPs, and their polymorphisms can be identified through PCR amplification [24,25,26,27].

In this study, we designed specific primers for amplifying *badh2-E2* and *badh2-E7* in different types of aromatic rice varieties and identified the characteristics of *badh2* gene mutations using Sanger sequencing. Based on the sequencing results of the *badh2* gene, multiplex-ready PCR and KASP molecular markers were designed for genotyping analysis and marker-assisted selection of the *badh2* gene, respectively. A recombinant inbred line (RILs) population was constructed by crossing the aromatic rice line Yexiang with the conventional non-aromatic rice line Yunongsimiao. Using KASP molecular markers, 98 homozygous aromatic lines were detected among 384 RILs. Through the pedigree method combined with 2-AP detection, an aromatic rice line, Zhexiangzhenhe, with aroma and excellent agronomic traits was bred.

## 2. Materials and Methods

### 2.1. Plant Materials

To identify the mutation types in the *badh2-E2* and *badh2-E7* loci among aromatic rice varieties currently promoted in production, this study selected eight aromatic *japonica* rice lines, sixteen aromatic *indica* rice lines, and two conventional non-aromatic rice lines (Table 1). All rice materials were planted in the summer season of 2022 in a paddy field at the Yangdu Experimental Base of the Zhejiang Academy of Agricultural Sciences, Haining County, China. Yexiang is an aromatic *indica* rice line with excellent taste and cooking quality, while Yuenongsimiao is a non-aromatic *indica* line characterized by high yield and strong adaptability to diverse environmental conditions. The F_1_ plants were generated from a cross between Yexiang (as the female parent) and Yuenongsimiao (as the male parent). Approximately 500 F_2_ seeds from this cross were sown. In each subsequent generation, around 50 plants with desirable agronomic traits were selected and self-pollinated until the F_7_ generation. A total of 384 plants were then genotyped using a functional KASP marker targeting the *badh2* gene. Ninety-eight F_8_ RILs with homozygous *badh2* genotypes were planted in the summer season of 2022 for phenotypic and yield-related observations. An elite line named Zhexiangzhenhe was selected to measure the 2-AP content, and phenotyping was conducted to determine the yield trait.

**Figure 1:**
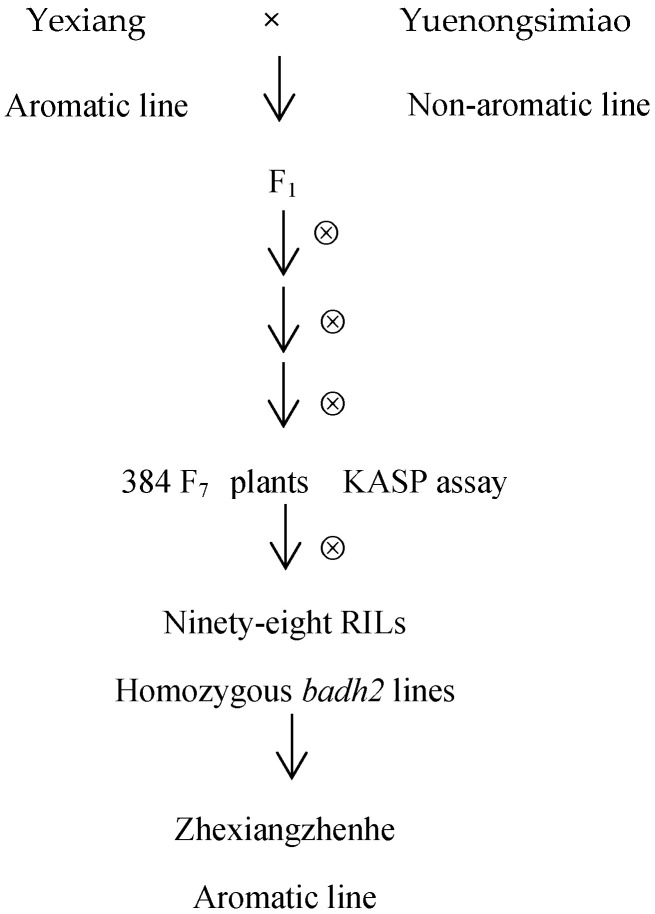
Selection scheme of the aromatic line of ZhexiangZhenhe via KASP.

The procedures are presented in Figure 1 in this study.

### 2.2. DNA Isolation and Marker Development

Genomic DNA was extracted from the leaf with a length of 1–2 cm using the CTAB buffer at the booting stage [28]. The DNA concentration was measured using a Nanodrop onec-1000 spectrophotometer (Thermo Fisher Scientific, Waltham, MA, USA) and adjusted to a working concentration of 100 ng/μL. To identify the deletion mutations at the *badh2-E2* and *badh2-E7* loci of the aromatic rice lines, specific primers were designed at positions 200–300 bp upstream and downstream of the deletion sites, respectively (Table 2). Based on the DNA sequence of the rice *Oryza sativa* cv. Nipponbare, a 554 bp fragment at the *badh2-E2* locus and a 442 bp fragment at the *badh2-E2* locus were amplified for Sanger sequencing analysis, respectively. According to the mutation type of eight aromatic *japonica* rice lines, a specific primer was designed for multiplex-ready PCR (Table 2). A 234 bp fragment for the aromatic line and a 241 bp fragment for the non-aromatic line were amplified for multiplex-ready PCR analysis. PCR reactions were prepared following the standard protocol for AmpliTaq DNA polymerase (Applied Biosystems, Foster City, CA, USA), with primer concentrations optimized for target amplification. The PCR amplification conditions were as follows: an initial denaturation step at 94 °C for 5 min, followed by 30 cycles of denaturation at 94 °C for 30 s, annealing at 56 °C for 45 s, and extension at 72 °C for 45 s. This was then followed by 8 additional cycles of 94 °C for 30 s (denaturation), 53 °C for 45 s (annealing), and 72 °C for 45 s (extension); finally, a final extension step was performed at 72 °C for 10 min. Subsequently, 1 µL of the PCR product was mixed with 22 µL of formamide and 0.5 µL of ROX standard (ABI), and the mixture was analyzed using an ABI 3730XL Prism Genetic Analyzer. The assay method for multiplex-ready PCR analysis was performed as previously described [27]. To design KASP markers for genotyping, approximately 200 bp flanking sequences (both upstream and downstream) surrounding the SNP locus of exon 7 of the *badh2* gene were analyzed. KASP primers were then designed following standard KASP guidelines and synthesized by LGC Limited (Teddington, Middlesex, UK). The standard FAM (5′-GAAGGTGACCAAGTTCATGCT-3′) and HEX (5′-GAAGGTCGGAGTCAACGGATT-3′) fluorescent tags were incorporated and linked to allele-specific forward primers, each targeting the SNP at their respective 3′ ends. A common reverse primer was designed using the Primer 3 software [29], with the resulting amplification length restricted to less than 100 bp. The KASP assay method was performed as previously described [23].

### 2.3. Experiment Design and Measuring 2-AP Content

To observe phenotypic and agronomic traits, ninety-eight F_8_ RIls homozygous positive for *badh2* genes were planted in plots during the summer season in 2022. Each line was planted in 6 rows with 6 plants per row, maintaining a plant spacing and row spacing of 20 cm × 20 cm. Agronomic traits, including plant height, panicle length, number of filled grains per panicle, seed setting rate, and thousand-grain weight, were measured from 10 plants per line. Yuenongsimiao, an elite non-aromatic rice line, was included as a standard variety for comparison. Each agronomic trait measurement was conducted with three biological replicates, and each replicate included 10 plants of the same line to ensure the reliability of the data.

For the determination of 2-AP content, a volatile aromatic compound, 30 g of mature seeds was collected from the plants. After dehusking, the brown rice was ground into rice flour. Using 2,4,6-trimethylpyridine as the internal standard (concentration: 229.25 ng·ml-1), 400 mg of the rice flour sample was weighed in triplicate. The samples were placed in 10 mL narrow-mouth glass vials, and 0.8 mL of an ethanol extraction reagent containing the internal standard was added. The vials were incubated in an oven at 80 °C for 3 h for extraction. After extraction, the samples were taken out and allowed to cool to room temperature, then filtered through a 0.22 μm disposable syringe filter. A 150 μL aliquot of the filtrate was transferred into a liner tube, which was then placed in a 2 mL sample vial. The determination was performed using a GC-MS (gas chromatography-mass spectrometry) instrument (Shimadzu Corporation, Kyoto, Japan) [30]. The detection data were analyzed using the Excel 2010 and SPSS 17.0 software. The measurement of plant 2-AP content was performed at Huazhong Agricultural University, Wuhan, China.

### 2.4. Statistical Analysis

Statistical analyses were performed using SPSS 17.0 (IBM Corp., Armonk, NY, USA). Phenotypic differences between Zhexiangzhenhe and Yuenongsimiao were compared via one-way analysis of variance (ANOVA), followed by Tukey’s honestly significant difference (HSD) test for multiple comparisons.

## 3. Results

### 3.1. Identification of Mutation Type in Aromatic Lines

**Figure 2:**
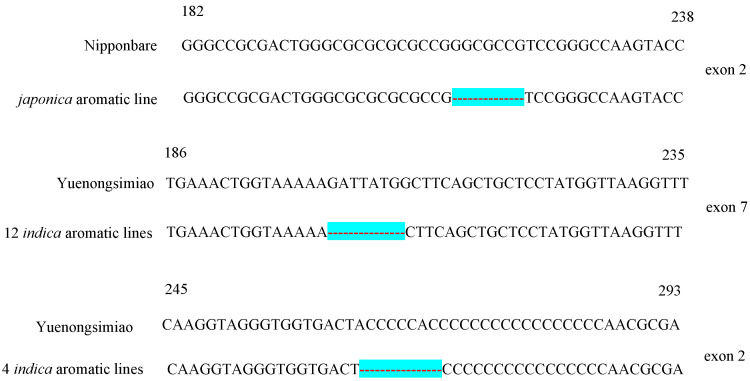
Mutation-type comparison of *badh2* alleles in different aromatic lines and non-aromatic lines. The blue-bottomed horizontal line indicates different base deletion fragments: 7 bp (5′-GGCGCCG-3′) deletion in *badh2-E2* of *japonica* aromatic lines, 8 bp (5′-GATTATGG-3′) deletion in *badh2-E7* of 12 *indica* aromatic lines, and 8 bp (5′-ACCCCCAC-3′) deletion in *badh2-E2* of 4 *indica* aromatic lines.

To clarify *badh2* mutation characteristics in currently cultivated aromatic rice, Sanger sequencing was performed on 24 aromatic lines targeting *badh2-E2* and *badh2-E7*. PCR amplification was performed on sixteen aromatic *indica* rice lines, eight aromatic *japonica* rice lines, and two non-aromatic lines using gene-specific primers. Sanger sequencing results revealed that the sixteen aromatic *indica* rice lines and eight aromatic *japonica* rice lines all harbor three distinct types of mutations in the *badh2-E2* and *badh2-E7* loci compared to two non-aromatic control lines. Among them, all eight *japonica* rice varieties exhibit a 7 bp (5′-GGCGCCG-3′) deletion in the *badh2-E2* locus, with consistent mutations across all aromatic *japonica* rice lines. However, sixteen aromatic *indica* rice lines have two mutation types in the *badh2* gene. Of these, twelve lines, including Nongxiang18, Nongxiang42, Yuzhenxiang, Chaungxiang5, Meixiangzhan2, Xiangyaxiangzhan, Yexiang, Zhexiangsimiao, 99xiang, Taixiang8, 19xiang, and Yingxiangsimiao, exhibit an 8 bp (5′-GATTATGG-3′) deletion in *badh2-E7*. Four lines, including Zhuxianglisi, Meixiangxinzhan, Junhexiangzhan, and Guangliangxiang3, exhibit an 8 bp (5′-ACCCCCAC-3′) deletion in *badh2-E2* (Figure 2). These results indicate that *badh2* mutations are subspecies-specific—7 bp deletion in *badh2-E2* for *japonica*, and two distinct deletions (8 bp in *badh2-E7* or *badh2-E2*) for *indica*.

### 3.2. Multiplex-Ready PCR Assay

**Figure 3:**
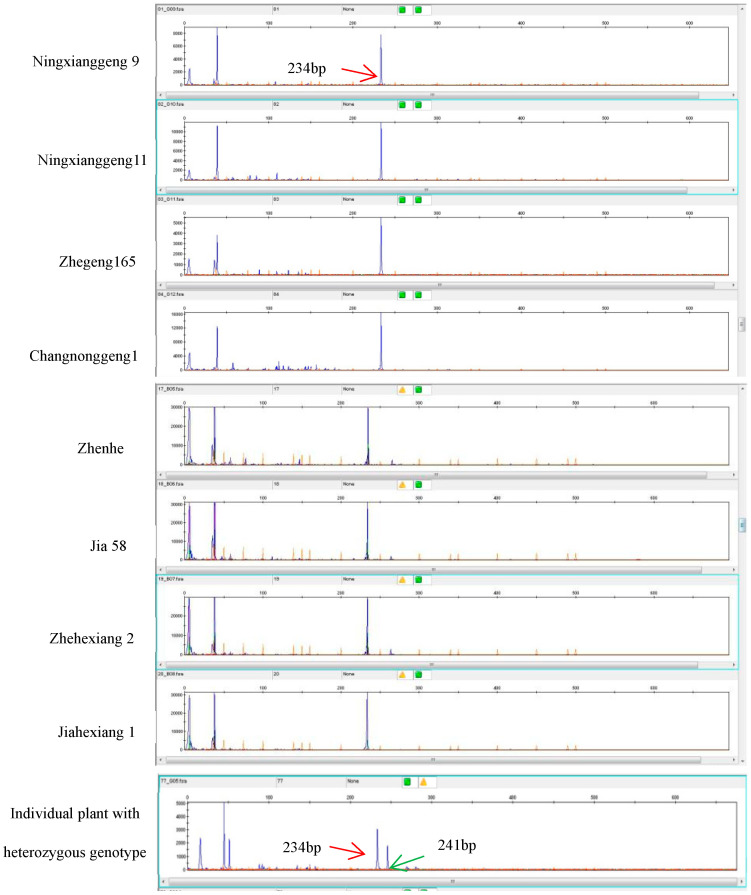
ABI 3730XL electrotraces showing multiplex-ready PCR amplified from eight aromatic *japonica* lines and individual plants with a heterozygous genotype. Individual plants with a homozygous *badh2* gene show only one fluorescent signal peak with a size of 234 bp, while individual plants with a heterozygous *badh2* gene exhibit two fluorescent signal peaks with sizes of 234 bp and 241 bp, respectively.

Based on the Sanger sequencing results of the *badh2* gene for eight *japonica* aromatic lines, we developed the marker for multiplex-ready PCR. Based on the fluorescence intensities from ABI 3730XL, a single fluorescent signal was detected in all eight japonica aromatic rice varieties, with a molecular size of 234 bp. To ensure the purity of aromatic rice varieties, 200 individual plants of the Jia 58 aromatic line were randomly selected, and multiplex-ready PCR analysis was performed on their *badh2* genes. The results showed that 199 individual plants had only one fluorescent signal peak, while the remaining plant had two fluorescent signal peaks, indicating that it might be a heterozygous plant, which was confirmed via phenotypic analysis (Figure 3). The multiplex-ready PCR assay exhibited high accuracy (99.5%) in genotyping, confirming its suitability for purification of aromatic rice varieties.

### 3.3. KASP Assay for the badh2 Gene

**Figure 4:**
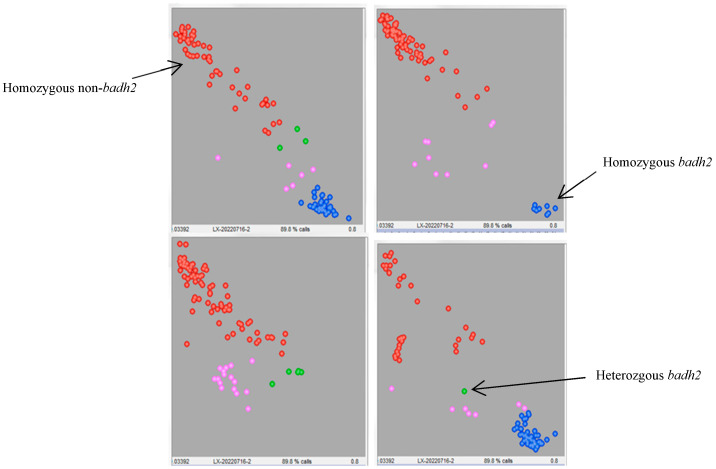
KASP assay of individual plants for the *badh2* gene in 384 F_7_ lines. The blue signal represents the homozygous genotype associated with aromatic traits; the red signal represents the homozygous genotype associated with non-aromatic traits; the green signal represents the heterozygous genotype, which also corresponds to non-aromatic traits; and the pink signal indicates genotypes that could not be determined.

To generate lines that possess both aromatic genes and excellent agronomic traits, genotyping analysis of the *badh2* gene was conducted on 384 F_7_ lines from the cross of Yexiang (aromatic) × Yuenongsimiao (non-aromatic) using KASP markers. The KASP assay for the *badh2* gene showed that ninety-eight individual plants were of the homozygous genotype with a blue signal, similar to Yexiang, while 238 individual plants were of the homozygous genotype with a red signal, similar to Yuenongsimiao, and thirty-nine individual plants were of the heterozygous genotype with a green signal. Unfortunately, the genotype of thirty-nine individual plants could not be determined with a pink signal (Figure 4).

### 3.4. Measurement of 2-AP Content and Agronomic Characteristic

**Figure 5:**
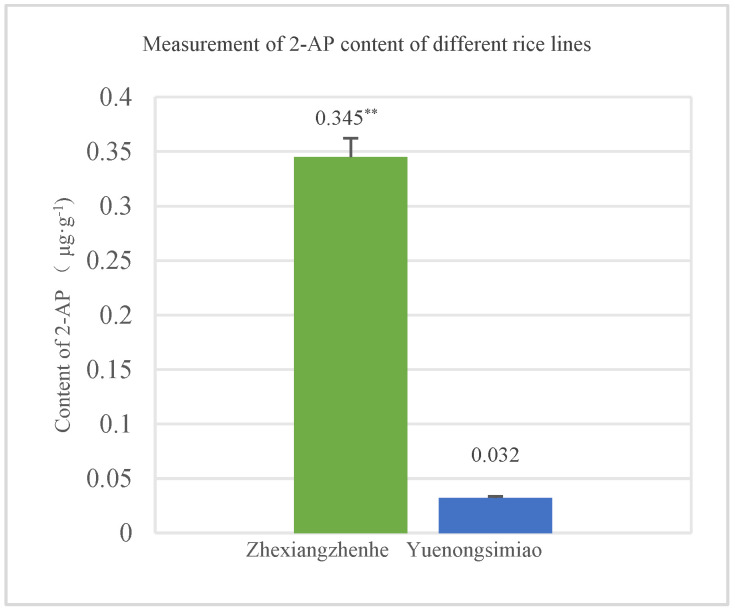
Measurement of 2-AP between Zhexiangzhenhe (aromatic line) and Yuenongsimiao (non-aromatic line). ** indicated that there are significant differences between different lines at the 0.01 level.

To determine the 2-AP content, mature seeds from Zhexiangzhenhe which possess homozygous *badh2* and Yuenongsimiao (non-aromatic) were selected and harvested. After dehusking, the seeds were ground into brown rice flour, and the 2-AP content was measured using a gas chromatography-mass spectrometry (GC-MS) system. The 2-AP content detection results showed that the average content in Zhexiangzhenhe was 0.345 μg·g^−1^, which was significantly higher than that in Yuenongsimiao, whose average content was only 0.032 μg·g^−1^ (*p* < 0.01) (Figure 5).

To assess differences in agronomic traits between Zhexiangzhenhe and Yuenongsimiao, statistical analyses were conducted on agronomic traits, including plant height, panicle length, effective panicles, total number of grains per panicle, number of filled grains per panicle, seed-setting rate, and 1000-grain weight (Table 3). The results revealed no significant differences between the two lines, indicating that Zhexiangzhenhe retained Yuenongsimiao’s agronomic traits while incorporating the aromatic gene *badh2*. This enables it to exhibit both aromatic rice quality and high-yield characteristics.

## 4. Discussion

As a staple food in regions such as East Asia, Southeast Asia, and South Asia, rice plays a crucial role in agricultural production. Aromatic rice, which is favored by consumers for its unique aroma, has higher commercial value compared to ordinary rice of the same category. Therefore, breeders have introduced aromatic genes to enable rice varieties to produce aromatic grains while retaining excellent agronomic traits. This not only increases agricultural benefits but also facilitates the promotion of aromatic rice varieties. 2-AP is the main component responsible for the aroma in aromatic rice. It can be detected in all tissues of aromatic rice varieties except roots [2]. A mutation in the *badh2* gene on rice chromosome 8 results in premature termination of aldehyde dehydrogenase translation. Consequently, the functionally conserved domain is not translated, rendering the enzyme incapable of catalyzing the dehydrogenation reaction. This leads to 2-AP loss, which is responsible for the characteristic aroma [3,13].

Different mutations in the *badh2* gene have been identified in some specific aromatic rice varieties. For example, Amarawathi et al. reported a new mutation in the *badh2* gene which has a 7 bp insertion in exon 8 in addition to an 8 bp deletion in *badh2-E7* [19]. Shao et al. reported an 803 bp deletion between exons 4 and 5, which was found in the aromatic variety Zaimiaoxiangnuo [31]. Shi et al. reported that in the aromatic rice variety Nankai 138, a 3 bp deletion was identified in the 5′ untranslated region of the *badh2* gene, accompanied by an 8 bp insertion in its promoter region [16]. However, the 8 bp deletion mutation in *badh2-E7* and the 7 bp deletion mutation in *badh2-E2* are mutation types widely found in most aromatic rice varieties [3,4,15,17]. In this study, the sequencing results of twenty-four aromatic rice lines showed that eight *japonica* aromatic rice lines had an 8 bp deletion mutation in the *badh2-E2* locus, which was highly conserved. Among the sixteen *indica* aromatic rice lines, twelve had a 7 bp deletion mutation in the *badh2-E7* locus, which was the same as the mutation in aromatic rice such as Basmati [3,17]. However, an 8 bp deletion mutation was identified in the *badh2-E2* among the other four *indica* aromatic rice lines, and this mutation differs from those found in aromatic rice varieties [3,16,17,19,31]. Meanwhile, no other mutations were detected in *badh2-E2* or other positions of *badh2-E7*, suggesting that this could potentially be a new type of mutation. This new mutation enriches the genetic variation library of the badh2 gene and provides a new molecular marker for the selection of aromatic traits in indica rice.

As an important molecular breeding technology, molecular marker-assisted selection has played a significant role in the research on improving the aromatic traits of rice. Specifically, the molecular marker techniques developed based on PCR and restriction enzyme digestion have been widely used in aromatic quality improvement [14,16,18,20,21,22]. However, with the continuous development and optimization of fluorescent labeling technology, multiplex-ready PCR and KASP technologies, which were developed based on PCR and fluorescent labeling, have demonstrated advantages such as high throughput and speed in molecular breeding and trait improvement, leading to their further application [23,31,32,33]. In this study, based on the 7 bp deletion in *badh2-E2* in eight *japonica* aromatic rice varieties, we developed a multiplex-ready PCR technology to perform genotyping analysis of the *badh2* gene in these lines. Additionally, we screened 200 individual plants from the Jia 58 aromatic rice line and successfully removed one heterozygous plant, providing technical support for the purification and rejuvenation of aromatic rice varieties. In this study, based on the 8 bp deletion in *badh2-E7* identified in the Yexiang aromatic line, we developed KASP markers. These markers were used to screen 384 F_7_ plants derived from the cross between Yexiang and Yuenongsimiao. Through genotyping, ninety-eight F_8_ plants with homozygous *badh2* were obtained. Combined with phenotypic observations, an elite line named Zhexiangzhenhe was selected, which exhibited excellent agronomic traits and retained the aromatic phenotype. Analyzing the 2-AP content revealed that Zhexiangzhenhe exhibited significantly higher 2-AP levels than the non-aromatic line Yuenongsimiao, while its agronomic traits showed no significant differences compared to Yuenongsimiao. Thus, KASP markers enable accurate target gene selection and facilitate high-throughput genotyping in large-scale breeding populations, thereby providing breeders with a rapid and efficient molecular marker-assisted selection tool to accelerate target trait improvement.

This study developed multiplex-ready PCR and KASP markers for the *badh2* gene, which have been verified in specific rice materials, but there are still some limitations. First, the transferability of the developed markers across different breeding backgrounds needs to be further verified. At present, the markers are mainly validated in *japonica* and *indica* rice in China. Whether they can be effectively applied to other rice subspecies (such as aromatic rice in South Asia) remains to be tested, as differences in genetic background may affect the amplification efficiency and genotyping accuracy of the markers. Secondly, the current study only focused on the mutation of the *badh2* gene, but the aroma of rice is a complex quantitative trait, which may be regulated by other genes and environmental factors [34]. Therefore, further research on the interaction between *badh2* and other genes, as well as the influence of environmental factors on 2-AP synthesis, is needed to comprehensively understand the genetic mechanism of rice aroma.

## 5. Conclusions

This study identified three subspecies-specific *badh2* mutation types in 24 aromatic rice lines: a 7 bp deletion in *badh2-E2* (*japonica*), an 8 bp deletion in *badh2-E7* (75% of *indica*), and a potential novel 8 bp deletion in *badh2-E2* (25% of *indica*). Two high-throughput detection markers were developed: multiplex-ready PCR for accurate *badh2-E2* genotyping (suitable for variety purification) and KASP for high-throughput *badh2-E7* screening (suitable for large breeding populations). Both markers were validated to be reliable—enabling efficient selection of aromatic genotypes and overcoming the limitations of traditional aroma detection methods. Using KASP markers, we successfully bred the elite aromatic line Zhexiangzhenhe, which combines high 2-AP content (0.345 μg·g^−1^) and superior agronomic traits, providing a valuable germplasm resource for aromatic rice breeding. The marker system and genetic insights from this study lay a foundation for accelerating aroma trait improvement in rice.

## Figures and Tables

**Table 1:** Twenty-four aromatic and two non-aromatic rice lines were selected in this study.

Subspecies	Line Name	Aromatic Type
*japonica*	Ningxianggeng 9, Ningxianggeng 11, Zhegeng 165, Changnonggeng 1, Zhenhe, Jia 58, Jiahexiang 1	aromatic
*indica*	Zhuxianglisi, Meixiangxinzhan, Junhexiangzhan, Guangliangxiang 3, Nongxiang 18, Nongxiang 42, Yuzhenxiang, Chuangxiang 5, Meixiangzhan 2, Xiangyaxiangzhan, Yexiang, Zhexiangsimiao, 99 xiang, Taixiang 8, 19 xiang, Yingxiangsimiao	aromatic
*japonica*	Nippornbare	Non-aromatic
*indica*	Yuenongsimiao	Non-aromatic

**Table 2:** Primers for sequence analysis and marker development in this study.

Target Area	Primer	Primer Sequence	Usage
*Exon2*	exon2-F1	CACCCTCTGCTTCTGCCTCT	Sanger sequencing
	exon2-R1	CAGCCATGCTTCCAACTTATTC	
*Exon7*	exon7-F1	TGGTCTTCCTTCAGGTGTGC	Sanger sequencing
	exon7-R1	TCCAGTGAAACAGGCTGTCA	
*Exon2*	M13-Bh2-1F	TGTAAAACGACGGCCAGTCATCGGTACCCTCCTCTTCA	Multiplex-Ready PCR
	FAM-M13-Bh2-1F	FAM-TGTAAAACGACGGCCAGTCATCGGTACCCTCCTCTTCA	
	Bh2-1R	ATTGCGCGGAGGTACTTG	
*Exon7*	Kexon7-F1	GAAGGTGACCAAGTTCATGCTAAGGTAGGGTGGTGACTA	KASP assay
	Kexon7-F2	GAAGGTCGGAGTCAACGGATTAAGGTAGGGTGGTGACTC	
	Kexon7-R	CCTGTACGGAACACACGCA	

M13 primer: TGTAAAACGACGGCCAGT, FAM-labeled primer: FAM-TGTAAAACGACGGCCAGT, FAM-probe: GAAGGTGACCAAGTTCATGCT, and ROX-probe: GAAGGTCGGAGTCAACGGATT.

**Table 3:** Agronomic traits comparison between Zhexiangzhenhe and Yuenongsimiao.

Lines	PH	PL	EP	TNGP	NFGP	SSR	TGW (g)
Zhexiangzhenhe	115.7 ± 2.3	20.5 ± 3.1	305.8 ± 25.4	185.3 ± 5.6	165.7 ± 11.6	89.4 ± 3.8	24.2 ± 0.6
Yuenongsimiao	116.5 ± 3.5	20.3 ± 3.5	308.4 ± 28.8	183.8 ± 4.5	166.8 ± 12.5	90.75 ± 4.5	23.9 ± 0.5

Data are the means and SD, *p* > 0.05, Student *t*-test.

## Data Availability

The data presented in this study are available upon request from the corresponding author.

## Abbreviations

The following abbreviations are used in this manuscript:

2-AP	2-acetyl-1-pyrroline
CAPS	Cleaved amplified polymorphism sequence
dCAPS	Derived CAPS
HRM	High-resolution melting
TS-PCR	Temperature switch PCR
KASP	Kompetitive allele-specific PCR
*badh2-E2*	The second exon of *badh2*
*badh2-E7*	The seventh exon of *badh2*
SNP	Single nucleotide polymorphism
RILs	Recombinant inbred lines
PH	Plant height
PL	Panicle length
EP	Effective panicles 10^4^/ha
TNGP	Total number of grains per panicle
NFGP	Number of filled grains per panicle
SSR	Seed sating rat
TGW	Thousand-grain weight (g)
